# Supplementary material for: Targeting P2Y14R protects against necroptosis of intestinal epithelial cells through PKA/CREB/RIPK1 axis in ulcerative colitis
Source: Nat Commun. 2024 Mar 7;15:2083. doi: 10.1038/s41467-024-46365-x (PMC10920779; doi:10.1038/s41467-024-46365-x)
Supplement: Supplementary file 1 — Supplementary Information [file 41467_2024_46365_MOESM1_ESM.pdf]

Supplementary Information for

Targeting P2Y$_{14}$R protects against necroptosis of intestinal epithelial cells through PKA/CREB/RIPK1 axis in ulcerative colitis

Chunxiao Liu [1], Hui Wang [2], Lu Han [1], Yifan Zhu [2], Shurui Ni [1], Jingke Zhi [1], Xiping Yang [1], Jiayi Zhi [1], Sheng Tian [2], Huanqiu Li [2, *], Qinghua Hu [1, *]

Correspondence to: huqh@cpu.edu.cn

This PDF file includes:

Supplementary Figures 1 to 12, Supplementary Tables 1 to 2, Spectral data and Chemical synthesis and structural characterization of the target compounds

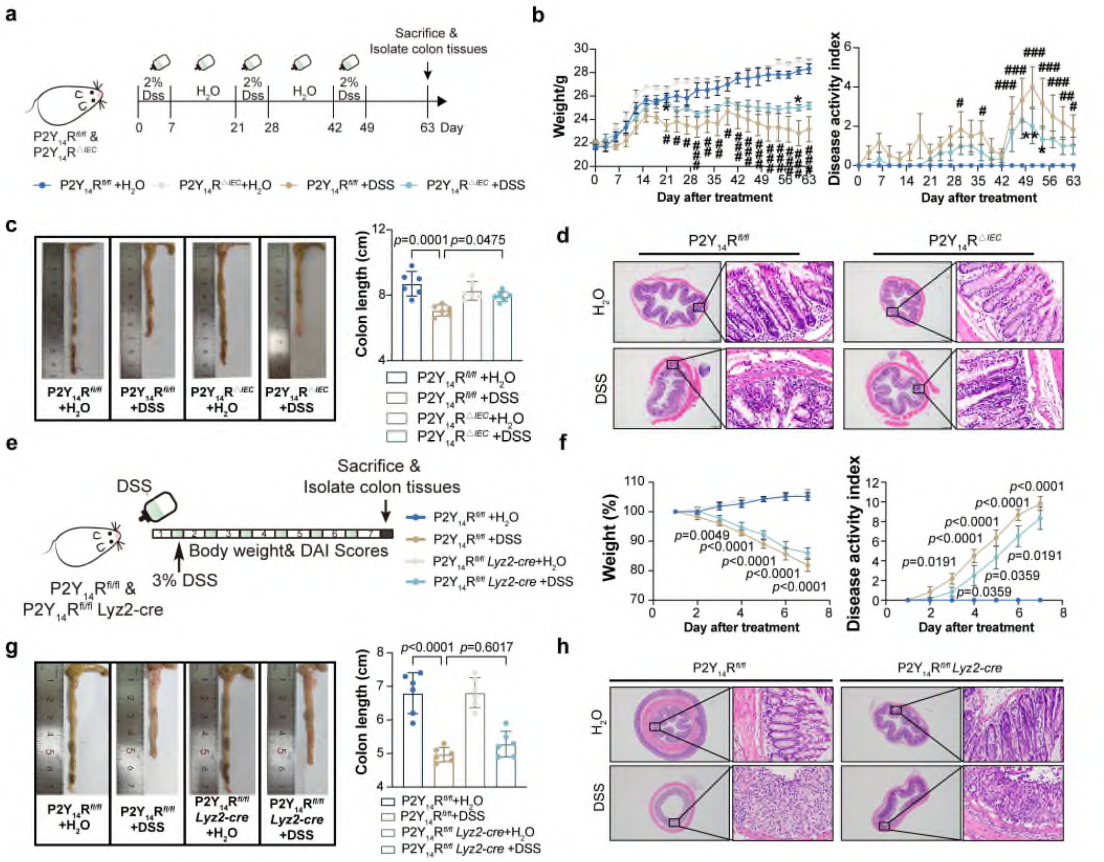

Supplementary Figure 1. The effect of P2Y$_{14}$R expressed by intestinal epithelial cell in regulating DSS-induced chronic experimental colitis and The effect of P2Y$_{14}$R expressed by myeloid cells in regulating DSS-induced experimental colitis.

a. Experimental Flow Chart. **b.** Body weight change and disease activity index evaluation of mice change during the disease process (n = 6 mice per group), the exact *p*-value is provided in the raw data. **c.** The length of colons from mice after DSS treatment (n = 6 mice per group). **d.** The H&E staining in the colon tissues of DSS-treated mice (Scale bar = 200 μm). **e.** Experimental Flow Chart. **f.** Body weight change and DAI evaluation during the disease process (n = 6 mice per group). **g.** The length of colons from P2Y$_{14}$R$^{fl/fl}$ and P2Y$_{14}$R$^{fl/fl}$ *Lyz2-cre* mice after DSS treatment (n = 6 mice per group). **h.** The H&E staining in colon tissues of DSS-treated mice (Scale bar = 200 μm). The data represent the mean ± SD for c and g, the data represent the mean ± SEM for b and f. The *p*-values were determined by two-way analysis of variance (ANOVA) with Šídák's multiple comparisons test for **b and f,** and One-way analysis of variance (ANOVA) with Tukey multiple comparison test for **c and g**. For **d** and **h**, each image was acquired independently three times, with similar results. Source data are provided as a Source Data file.

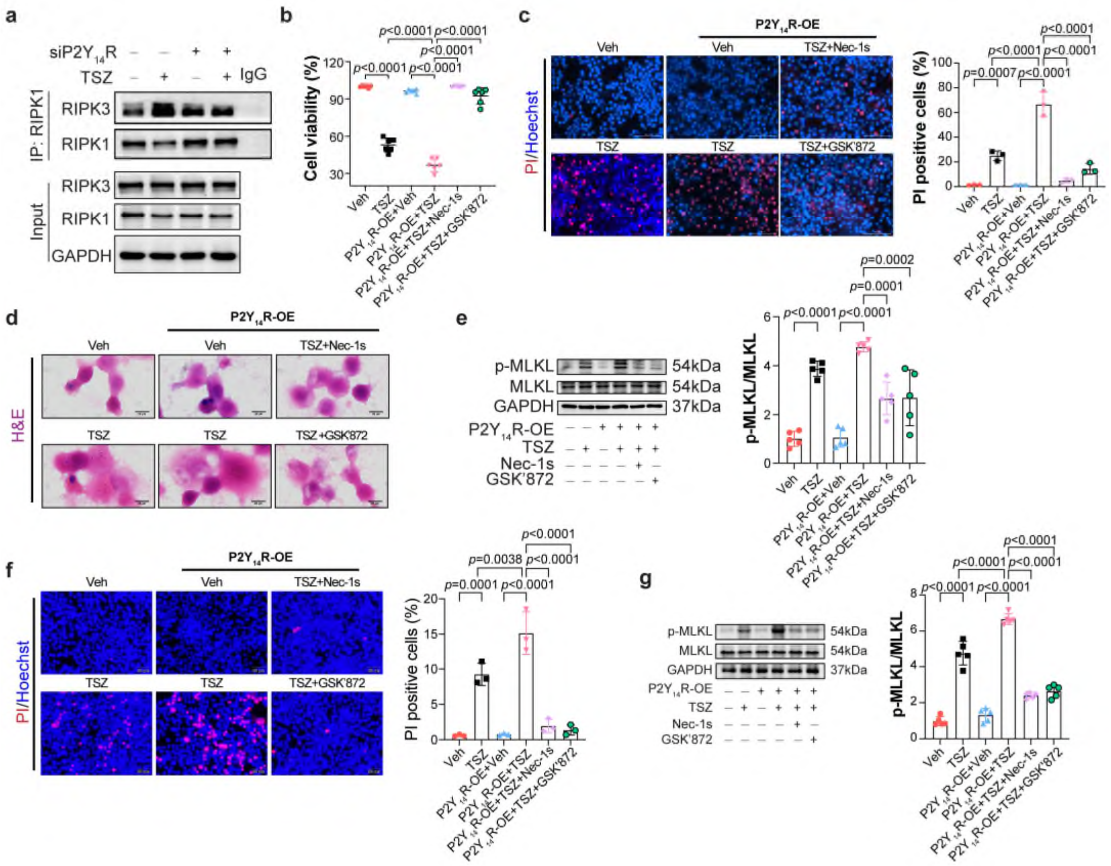

Supplementary figure 2. P2Y$_{14}$R regulates necroptosis of IECs via RIPK1/RIPK3 pathway.

a The interaction between RIPK1 and RIPK3 were determined by co-immunoprecipitation assays with IgG used as a control. GAPDH was used as a loading control. For **b** to **e**, The plasmid of human P2Y$_{14}$R and its control were used to transfect HT-29 cells with Lipofectamine 2000 for 48 h. 1h pre-treatment of Nec-1s and GSK’872 were exposed to HT-29 cells followed by TSZ model for 8 h. **b** Cell viability was determined by CCK8 analysis (n = 6 samples per group). **c** PI/Hoechst staining and PI positive cells analysis of HT-29 cells (n = 3 samples per group). **d** H&E cell staining, PI/Hoechst staining and PI positive cells analysis of HT-29 cells. **e** Phosphorylation of MLKL as well as its protein levels were analyzed by immunoblotting with corresponding antibodies in HT-29 cells (n = 5 samples per group). For **f** to **g**, The plasmid of human P2Y$_{14}$R and its control were used to transfect HCT-116 cells with Lipofectamine 2000 for 48 h. 1h pre-treatment of Nec-1s and GSK’872 were exposed to HT-29 cells followed by TSZ model for 8 h. **f** PI/Hoechst staining and PI positive cells analysis of HCT-116 cells (n = 3 samples per group). **g** Phosphorylation of MLKL

as well as its protein levels were analyzed by immunoblotting with corresponding antibodies in HCT-116 cells (n = 5 samples per group). The data represent the mean ± SD, and statistical significance was determined by One-way ANOVA with Tukey multiple comparison test. For **a** and **d**, each image was acquired independently three times, with similar results. For **a** and **d**, each image was acquired independently three times, with similar results. Source data are provided as a Source Data file.

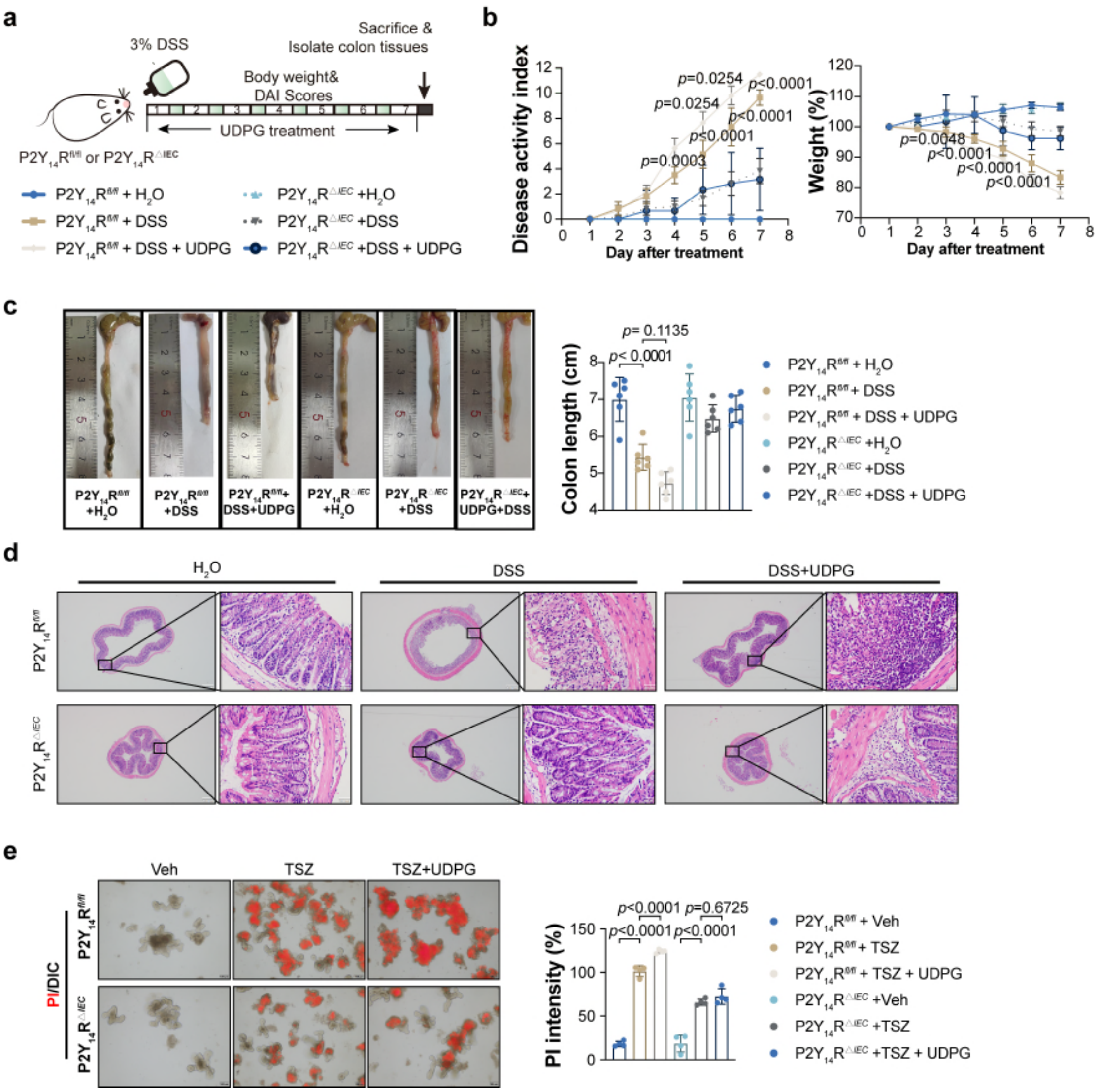

Supplementary figure 3. UDP-glucose aggravated DSS induced experiment colitis through targeting P2Y$_{14}$R.

a Experimental Flow Chart. **b** Body weight change and DAI evaluation during the disease process (n = 6 mice per group). **c** The length of colons from P2Y$_{14}$R$^{fl/fl}$ and P2Y$_{14}$R$^{\Delta IEC}$ mice after DSS

treatment (n = 6 mice per group). **d** The H&E staining in colon tissues of DSS-treated mice (Scale bar = 200 μm). e The PI staining and quantification of intestinal organoids from $P2Y_{14}R^{fl/fl}$ and $P2Y_{14}R^{\triangle IEC}$ mice treated as indicated with Veh, TSZ and TSZ + UDPG (n = 4 mice per group). The data represent the mean ± SD for **c** and **e**, the data represent the mean ± SEM for **b**. The *p*-values were determined by two-way analysis of variance (ANOVA) with Šídák's multiple comparisons test for **b**, and One-way analysis of variance (ANOVA) with Tukey multiple comparison test for **c**. For **d**, each image was acquired independently three times, with similar results. Source data are provided as a Source Data file.

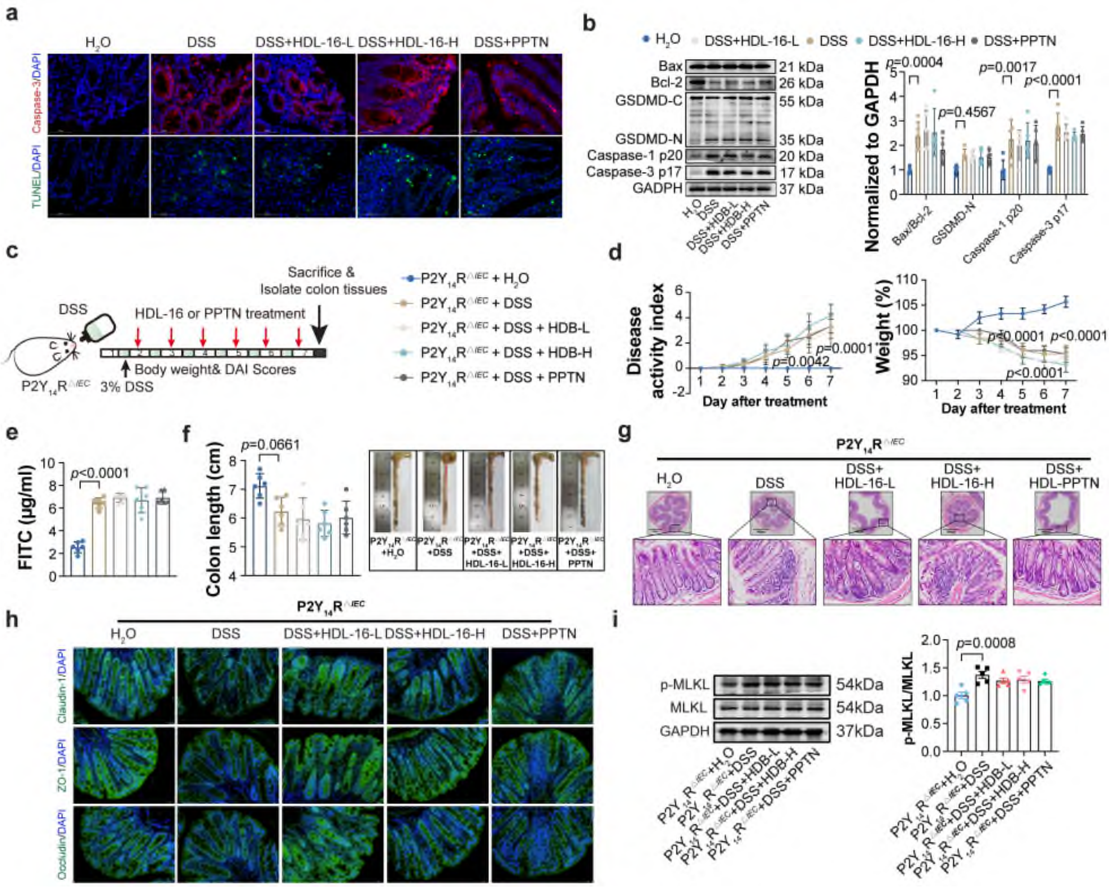

Supplementary figure 4. HDL-16 improved DSS-induced colitis through targeting $P2Y_{14}R$.

a The TUNEL staining of colon tissues from mice with HDL-16 or PPTN administration. The immunofluorescent images of colon tissues stained with cleave caspase-3 (Scale bar = 50 μm). **b** The expression of Bax, Bcl-2, GSDMD, caspase-1 p20 as well as caspase-3 p17 were analyzed by

immunoblotting with corresponding antibodies in the IECs from DSS treated mice with HDL-16 or PPTN administration (n = 5 mice per group). **c** Experimental Flow Chart, $P2Y_{14}R^{\triangle IEC}$ mice exposed to 3% DSS intraperitoneally received Low-dose HDL-16, High-dose HDL-16, or PPTN throughout the entire experimental period. **d** Body weight change and disease activity index evaluation of mice change during the disease process (n = 6 mice per group). **e** Effects of $P2Y_{14}R$ antagonists on $P2Y_{14}R^{\triangle IEC}$ mice mucosal barrier function as measured by serum levels of FITC-dextran based on intestinal permeability methods (n = 6 mice per group). **f** The length of colons from mice 7 days after DSS treatment (n = 6 mice per group). **g** The H&E staining in the colon tissues of DSS-treated mice (Scale bar = 200 μm). **h** The immunofluorescent images of colon tissues stained with Claudin-1, Occludin and ZO-1, the principal components of tight junction (Scale bar = 200 μm). **i** Phosphorylation MLKL as well as its protein levels were analyzed by immunoblotting with corresponding antibodies in colon tissues (n = 5 mice per group). The data represent the mean ± SD for **b**, **e**, **f** and **i**, the data represent the mean ± SEM for **d**. The *p*-values were determined by One-way analysis of variance (ANOVA) with Tukey multiple comparison test for **e**, **f**, **i**, and two-way analysis of variance (ANOVA) with Šídák's multiple comparisons test for **b** and **d**. For **a, g** and **h**, each image was acquired independently three times, with similar results. Source data are provided as a Source Data file.

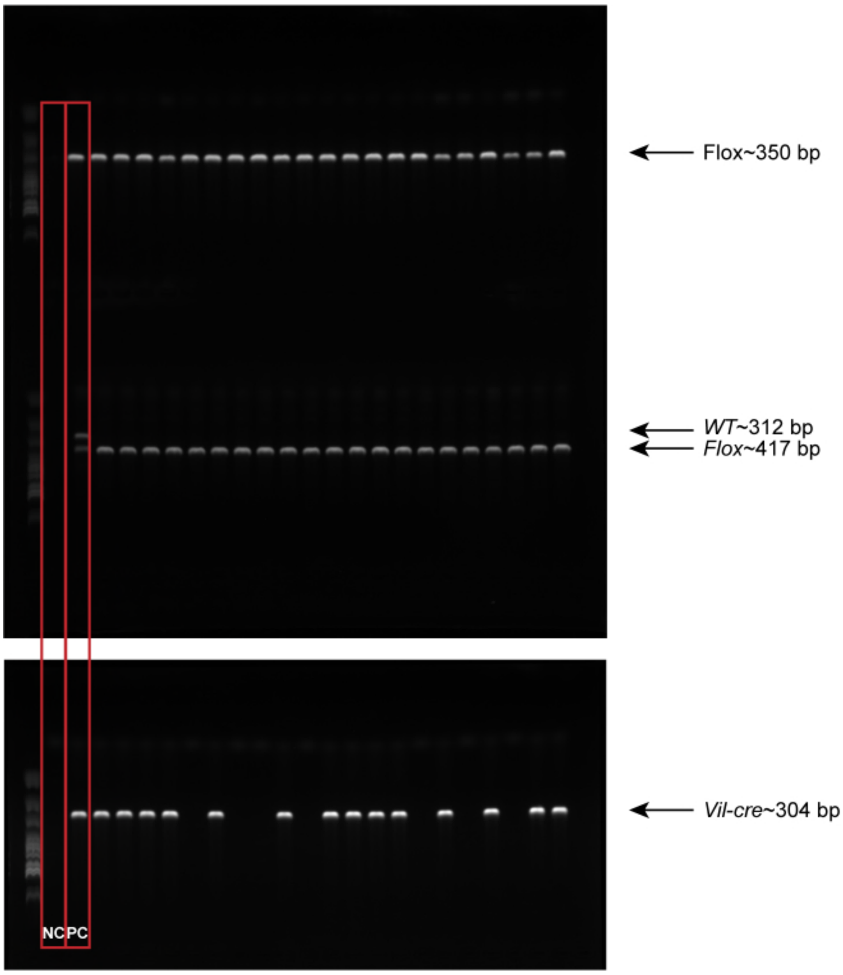

Supplement figure 5. Representative gene identification results of experimental mice.
PC represents a positive control and NC represents a negative control. Source data are provided as a Source Data file.

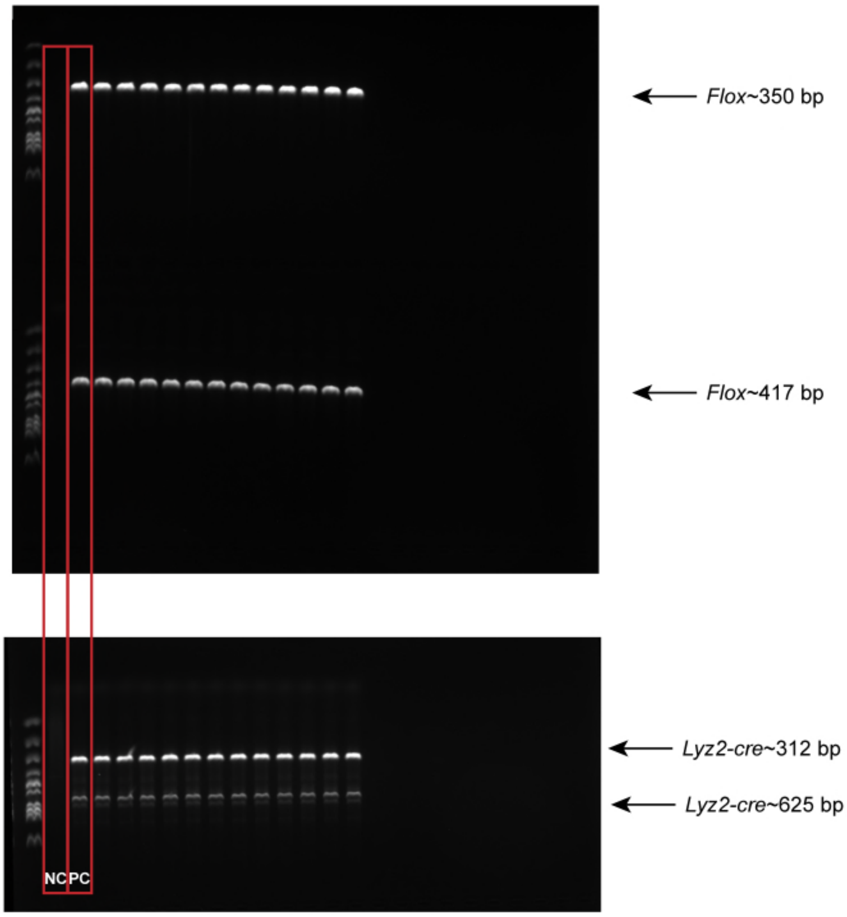

Supplement figure 6. Representative gene identification results of experimental mice.
PC represents a positive control and NC represents a negative control. Source data are provided as a Source Data file.

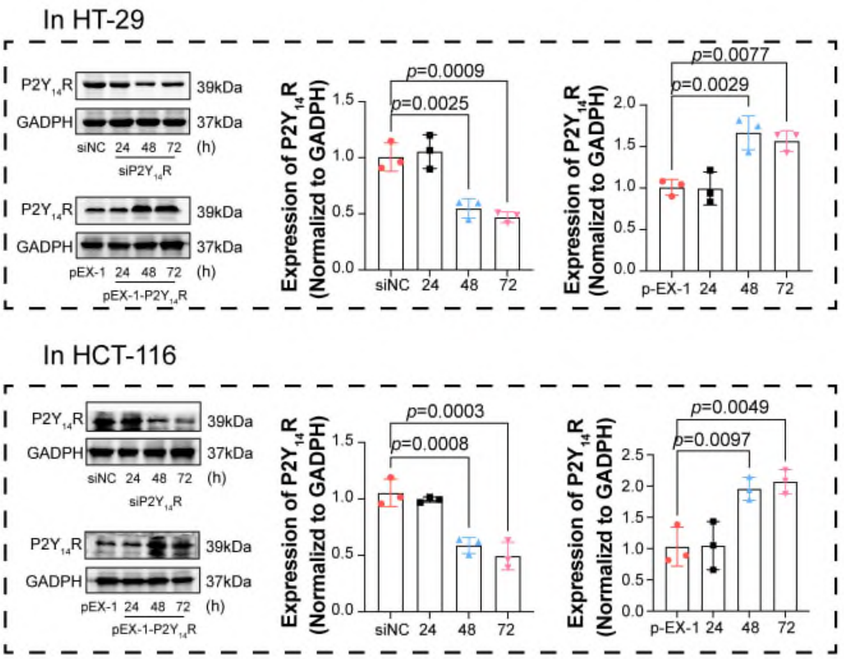

Supplementary figure 7. Transfection efficiency of overexpression constructs and siRNA knockdown in HT-29 and HCT-116 cells.

The data represent the mean ± SD of three independent experiments, with similar results. The *p*-values were determined by One-way analysis of variance (ANOVA) with Tukey multiple comparison test. Source data are provided as a Source Data file.

Supplementary Table 1. RT-PCR primers

Name	Species	Application	Forward primer	Reverse primer
Ripk1	Mouse	RT-PCR	GGTCAAATTCAGAACAACCTGGA	CACACTGCGATCATTCTCGT
Ugp2	Mouse	RT-PCR	AGGTCCGCCTCTCCATTGAT	AAACTTGGTGGGACCGACTC
Gys1	Mouse	RT-PCR	GCTGAGAGGGATCGGCTAAATA	GGGCAGCTCATTTTCTTGTG
P2ry14	Mouse	RT-PCR	AGCAGATCATTCCCGTGTTGT	AGCCACCACTATGTTCTTGAGA
Gapdh	Mouse	RT-PCR	AGGTCGGTGTGAACGGATTTG	TGTAGACCATGTAGTTGAGGTCA
Ripk1	Human	RT-PCR	GGGAAGGTGTCTCTGTGTTTC	CCTCGTTGTGCTCAATGCAG
Gapdh	Human	RT-PCR	GAAGGTGAAGGTCGGAGTCAAC	CATCGCCCCACTTGATTTTGGA
Ripk1 promoter-site1		CHIP	TAATTCAGGCCCTCAGGAGA	GTGTAAGCAGCCCCATTTTC
Ripk1 promoter-site2		CHIP	CCACTGGAACAGGGAGCTT	AGATCCGTCTACCCCCAGTC

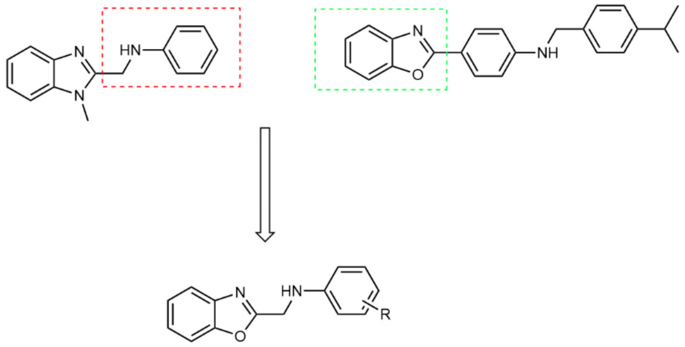

Supplementary figure 8. Design strategy of $P2Y_{14}R$ antagonists.

Rational optimisation of compounds obtained by virtual screening

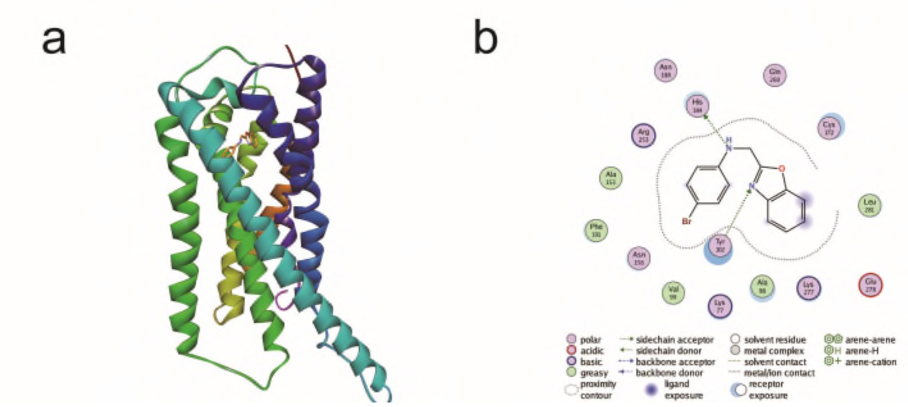

Supplementary figure 9. Binding modes of compound HDL-16 with P2Y$_{14}$R homology modeland ligand interaction diagram of compound HDL-16 with P2Y$_{14}$R complex structure. **a** The predicted conformations of compound HDL-16 derived from Glide docking. **b** The predicted interaction patterns for compound HDL-16 in the binding pocket

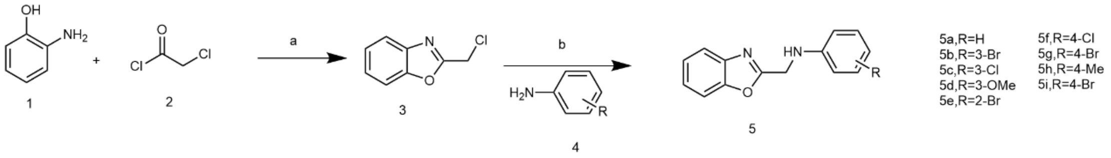

Supplementary figure 10. General synthetic route of P2Y$_{14}$R antagonists.

Reagents and conditions: Et3N(1.1 eq), xylene, overnight at 145°C（b）K2CO3(0.3 eq),KI(1.0 eq), ethanol, reflux 2h at 80°C.

Based on the synthetic route of optimized compounds **5a-i** as shown in supplementary Fig. 10, we prepared a total of 9 rational-designed compounds. Commercially available o-amino phenol (**1**) were used as raw materials, which underwent a cyclization reaction with chloroacetyl chloride (**2**) in xylene to generate the corresponding intermediate 2-(chloromethyl)benzo[d]oxazole (**3**).Compounds **5a-i** were obtained by the nucleophilic substitution of **3** and commercially available different substituted aniline (**4)** in the presence of K_2CO_3 and KI in DMF. The yield varied from 38% to 86%.

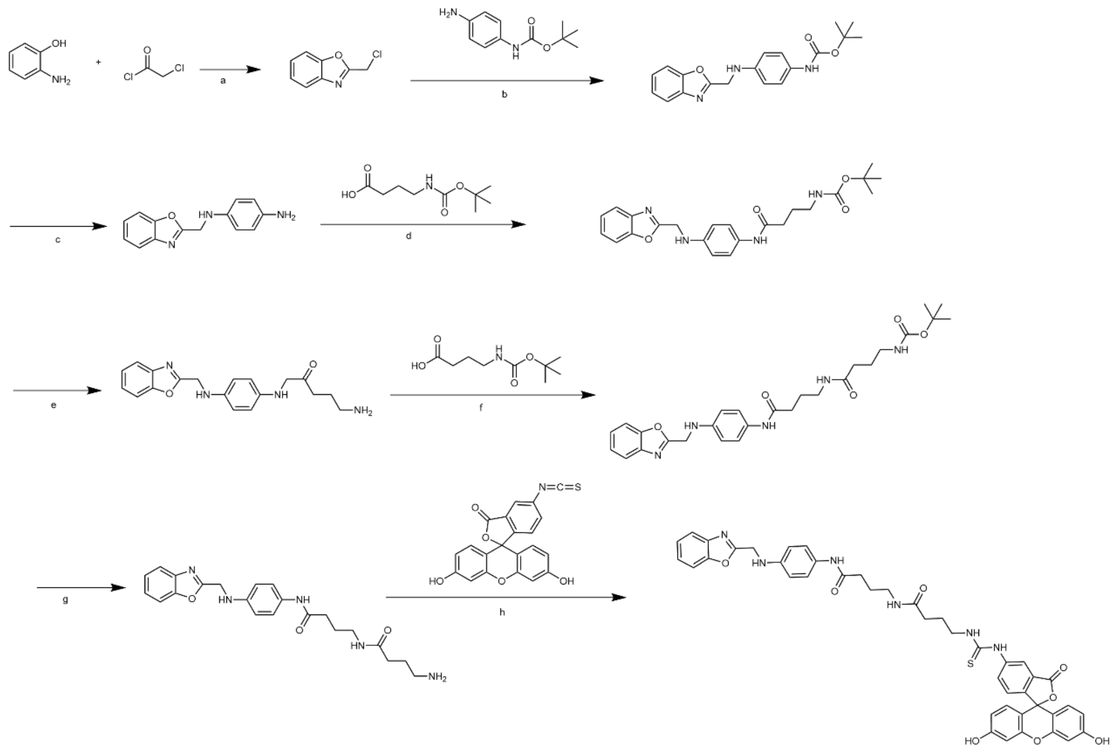

Supplementary figure 11. Synthesis of P2Y$_{14}$R Fluorescent Probe.

Reagents and conditions: (a) Chlorobenzene, p-Toluenesulfonic, Pyridine, rt- reflux 8h; (b) Ethanol, KI, 1 mol/L KOH aq, reflux, 8h (c) TFA, DCM rt, 1 h; (d) DMF, HOBT, EDCI, TEA, rt, 12 h; (e)TFA, DCM rt, 1 h; (f) DMF, HOBT, EDCI, TEA, rt, 12 h; (g)TFA, DCM rt, 1 h; (h) FITC, TEA, DMF, rt, 12 h.

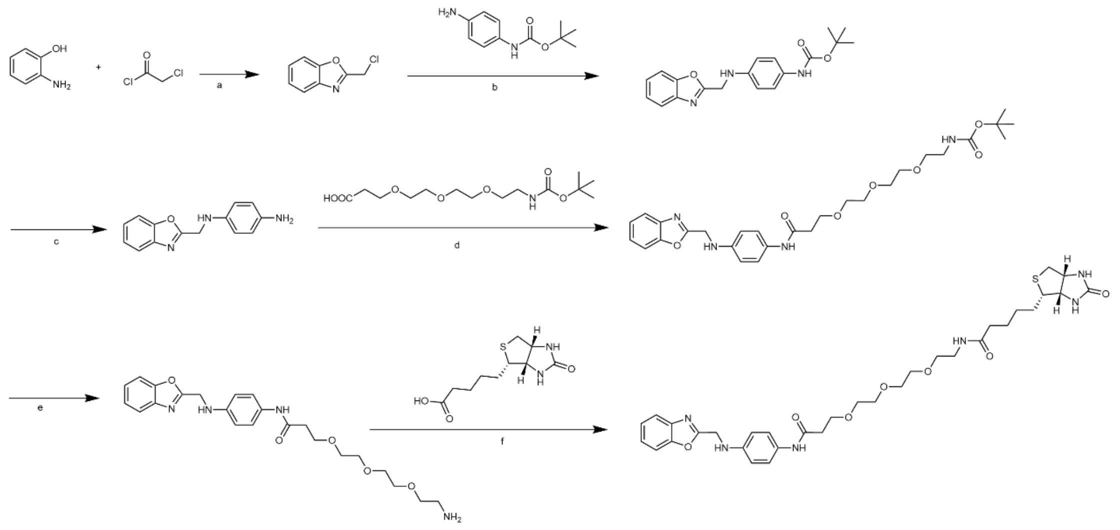

Supplementary figure 12. Synthesis of biotin-labeled affinity probe

Reagents and conditions: (a) Chlorobenzene, p-Toluenesulfonic, Pyridine, rt- reflux 8h; (b) Ethanol, KI, 1 mol/L KOH aq, reflux, 8h (c) TFA, DCM rt, 1 h; (d) DMF, HOBT, EDCI, TEA, rt, 12 h; (e)TFA, DCM rt, 1 h; (f) FITC, TEA, DMF, rt, 12 h.

Chemical synthesis and structural characterization of the target compounds

General. All reagents and solvents were commercially available and were used without further purification. Varian UNTIY INOVA 400 MHz and 600 MHz NMR instruments were used to

determine the hydrogen and carbon spectra of NMR. The silica gel used for separation of compound column chromatography (CC) was 200-300 mesh column chromatography silica gel produced by Qingdao Ocean Chemical Works. The amount of silica gel was 50-100 times of the amount of separated samples. The whole elution process was tracked by thin layer chromatography (TLC). The silica gel 60 GF254 produced by Qingdao Ocean Chemical Works was used for thin layer chromatography. The silica gel 60 GF254 was detected by ultraviolet radiation at 254 nm wavelength.

5a 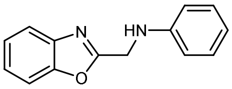

N-(benzo[d]oxazol-2-ylmethyl)aniline (5a)

A mixture of aniline (18.0 mmol) and 2-(chloromethyl)benzo[d]oxazole (18.0 mmol) was dissolved in DMF (10 mL). Potassium carbonate (0.90 g, 6.48 mmol) and KI was added to the mixture. The solution at room temperature was stirred for 12 hours. And then the solution was poured into water and extracted with ethyl acetate. The combined organic layers were washed with water and brine, dried over Na2SO4 and concentrated in vacuo. The residue was purified by column chromatography on silica gel (petroleum ether : ethyl acetate = 4:1) to give compound **5a**.

^{1}H NMR (600MHz, CDCl3) δ7 .71-7 .69(m, 1H), 7 .51-7 .48(m, 1H), 7 .33-7 .30(m, 2H), 7 .20(dd, J = 11 .8，4 .1Hz, 2H), 6 .79-6 .74(m, 3H), 4 .61(s, 2H).

^{13}C NMR (151MHz, CDCl3)δ164 .08, 150 .85, 146 .84, 140 .86, 129 .35, 129 .25, 125 .05, 124 .43, 119 .95, 118 .61, 115 .12, 113 .21, 110 .63, 77 .21, 77 .00, 76 .79, 41 .92.

5b 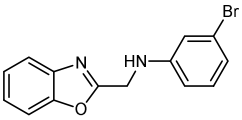

N-(benzo[d]oxazol-2-ylmethyl)-3-bromoaniline (5b)

A mixture of 3-methoxyaniline (18.0 mmol) and 2-(chloromethyl)benzo[d]oxazole (18.0 mmol) was dissolved in DMF (10 mL). Potassium carbonate (0.90 g, 6.48 mmol) and KI was added to the mixture. The solution at room temperature was stirred for 12 hours. And then the solution was poured into water and extracted with ethyl acetate. The combined organic layers were washed with water and brine, dried over Na2SO4 and concentrated in vacuo. The residue was purified by column chromatography on silica gel (petroleum ether : ethyl acetate = 4:1) to give compound **5b**.

^{1}H NMR (600MHz, DMSO-d6) δ7 .58(d, J = 7 .2Hz, 1H), 7 .42(d, J = 7 .4Hz, 1H), 7 .16-7 .08(m, 2H), 7 .00(t, J = 8 .0Hz, 1H), 6 .82(s, 1H), 6 .69(d, J = 7 .8Hz, 1H), 6 .61(s, 1H), 4 .48(d, J = 5 .7Hz, 2H).

^{13}C NMR (151MHz, DMSO-d6) δ153 .47, 150 .51, 135 .15, 122 .77, 121 .93, 120 .13, 118 .19, 116 .27, 114 .35, 113 .12, 111 .72, 41 .87, 40 .35, 40 .21, 40 .07, 39 .83, 39 .79, 38 .65, 35 .5.

5c 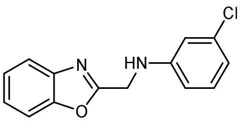

N-(benzo[d]oxazol-2-ylmethyl)-3-chloroaniline (5c)

A mixture of 3-chloroaniline (18.0 mmol) and 2-(chloromethyl)benzo[d]oxazole (18.0 mmol) was

dissolved in DMF (10 mL). Potassium carbonate (0.90 g, 6.48 mmol) and KI was added to the mixture. The solution at room temperature was stirred for 12 hours. And then the solution was poured into water and extracted with ethyl acetate. The combined organic layers were washed with water and brine, dried over Na2SO4 and concentrated in vacuo. The residue was purified by column chromatography on silica gel (petroleum ether : ethyl acetate = 4:1) to give compound **5c**.

^{1}H NMR (600MHz, DMSO-d6) δ7 .56(dd, J = 21 .3, 12 .6Hz, 2H), 7 .26(dd, J = 7 .8, 1 .2Hz, 1H), 7 .13(dd, J = 6 .0, 3 .1Hz, 2H), 7 .07(t, J = 7 .7Hz, 1H), 6 .67(d, J = 8 .2Hz, 1H), 6 .61(td, J = 7 .8, 1 .2Hz, 1H), 4 .60(d, J = 5 .8Hz, 2H).

^{13}C NMR (151MHz, DMSO-d6) δ156 .47, 150 .51, 133 .15, 122 .58, 120 .93, 119 .13, 118 .19, 116 .27, 115 .03, 113 .12, 111 .72, 41 .87, 40 .35, 40 .21, 40 .07, 39 .93, 39 .79, 39 .65, 39 .51.

5d 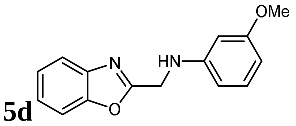

N-(benzo[d]oxazol-2-ylmethyl)-3-methoxyaniline (5d)

A mixture of 3-methoxyaniline (18.0 mmol) and 2-(chloromethyl)benzo[d]oxazole (18.0 mmol) was dissolved in DMF (10 mL). Potassium carbonate (0.90 g, 6.48 mmol) and KI was added to the mixture. The solution at room temperature was stirred for 12 hours. And then the solution was poured into water and extracted with ethyl acetate. The combined organic layers were washed with water and brine, dried over Na2SO4 and concentrated in vacuo. The residue was purified by column chromatography on silica gel (petroleum ether : ethyl acetate = 4:1) to give compound **5d**.

^{1}H NMR (400MHz, CDCl3) δ7 .74-7 .71(m, 1H), 7 .54-7 .51(m, 1H), 7 .34(dd, J = 6 .7, 3 .5Hz, 2H), 7 .32-7 .32(m, 1H), 6 .83-6 .80(m, 2H), 6 .77-6 .73(m, 3H), 4 .60(s, 2H), 3 .76(s, 3H).

^{13}C NMR (101MHz, CDCl3)δ164 .38, 152 .92, 150 .86, 140 .97, 140 .91, 125 .04, 124 .42, 119 .96, 116 .76, 114 .94, 114 .68, 110 .65, 77 .36, 77 .25, 77 .05, 76 .73, 55 .72, 42 .87, 29 .71.

5e 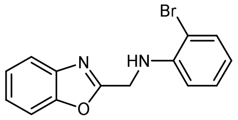

N-(benzo[d]oxazol-2-ylmethyl)-2-bromoaniline (5e)

A mixture of 2-bromoaniline (18.0 mmol) and 2-(chloromethyl)benzo[d]oxazole (18.0 mmol) was dissolved in DMF (10 mL). Potassium carbonate (0.90 g, 6.48 mmol) and KI was added to the mixture. The solution at room temperature was stirred for 12 hours. And then the solution was poured into water and extracted with ethyl acetate. The combined organic layers were washed with water and brine, dried over Na2SO4 and concentrated in vacuo. The residue was purified by column chromatography on silica gel (petroleum ether : ethyl acetate = 4:1) to give compound **5e**.

^{1}H NMR (600MHz, DMSO-d6) δ7 .56(d, J = 7 .2Hz, 1H), 7 .30(d, J = 7 .4Hz, 1H), 7 .16-7 .10(m, 2H), 7 .00(t, J = 8 .0Hz, 1H), 6 .82(s, 1H), 6 .69(d, J = 7 .8Hz, 1H), 6 .61(s, 1H), 4 .47(d, J = 5 .7Hz, 2H).

^{13}C NMR (151MHz, DMSO-d6) δ155 .26, 150 .51, 131 .15, 122 .67, 121 .93, 119 .33, 117 .50, 116 .37, 115 .33, 114 .21, 112 .72, 41 .60, 40 .83, 40 .21, 40 .07, 39 .88, 39 .79, 39 .75, 39 .55.

5f 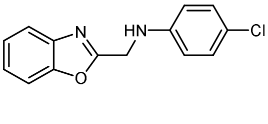

N-(benzo[d]oxazol-2-ylmethyl)-4-chloroaniline (5f)

A mixture of 4-chloroaniline (18.0 mmol) and 2-(chloromethyl)benzo[d]oxazole (18.0 mmol) was dissolved in DMF (10 mL). Potassium carbonate (0.90 g, 6.48 mmol) and KI was added to the mixture. The solution at room temperature was stirred for 12 hours. And then the solution was poured into water and extracted with ethyl acetate. The combined organic layers were washed with water and brine, dried over Na2SO4 and concentrated in vacuo. The residue was purified by column chromatography on silica gel (petroleum ether : ethyl acetate = 4:1) to give compound **5f**.

^{1}H NMR (600MHz, CDCl3) δ7 .69(dd, J = 6 .1, 3 .0Hz, 1H), 7 .50(dd, J = 6 .6, 2 .5Hz, 1H), 7 .34-7 .31(m, 2H), 7 .14(d, J = 8 .6Hz, 2H), 6 .67(d, J = 8 .7Hz, 2H), 4 .58(d, J = 5 .7Hz, 2H).

^{13}C NMR (151MHz, CDCl3) δ163 .63, 150 .84, 145 .81, 145 .38, 140 .76, 129 .37, 129 .19, 125 .16, 124 .51, 123 .32, 119 .98, 114 .31, 110 .64, 77 .20, 76 .99, 76 .78, 41 .95.

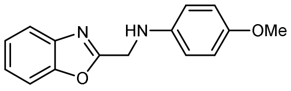

5g

N-(benzo[d]oxazol-2-ylmethyl)-4-methoxyaniline (5g)

A mixture of 4-methoxyaniline (18.0 mmol) and 2-(chloromethyl)benzo[d]oxazole (18.0 mmol) was dissolved in DMF (10 mL). Potassium carbonate (0.90 g, 6.48 mmol) and KI was added to the mixture. The solution at room temperature was stirred for 12 hours. And then the solution was poured into water and extracted with ethyl acetate. The combined organic layers were washed with water and brine, dried over Na2SO4 and concentrated in vacuo. The residue was purified by column chromatography on silica gel (petroleum ether : ethyl acetate = 4:1) to give compound **5g**.

^{1}H NMR (400MHz，CDCl3) δ7 .74-7 .71(m, 1H), 7 .54-7 .51(m, 1H), 7 .34(dd, J = 6 .7, 3 .5Hz, 2H), 7 .32-7 .32(m, 1H), 6 .83-6 .80(m, 2H), 6 .77-6 .73(m, 3H), 4 .60(s, 2H), 3 .76(s, 3H).

^{13}C NMR (101MHz, CDCl3) δ164 .38, 152 .92, 150 .86, 140 .97, 140 .91, 125 .04, 124 .42, 119 .96, 116 .76, 114 .94, 114 .68, 110 .65, 77 .36, 77 .25, 77 .05, 76 .73, 55 .72, 42 .87, 29 .71.

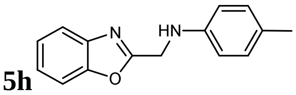

5h

N-(benzo[d]oxazol-2-ylmethyl)-4-methylaniline (5h)

A mixture of p-toluidine (18.0 mmol) and 2-(chloromethyl)benzo[d]oxazole (18.0 mmol) was dissolved in DMF (10 mL). Potassium carbonate (0.90 g, 6.48 mmol) and KI was added to the mixture. The solution at room temperature was stirred for 12 hours. And then the solution was poured into water and extracted with ethyl acetate. The combined organic layers were washed with water and brine, dried over Na2SO4 and concentrated in vacuo. The residue was purified by column chromatography on silica gel (petroleum ether : ethyl acetate = 4:1) to give compound **5h**.

^{1}H NMR (600MHz, CDCl3) δ7 .70(dt, J = 7 .6, 3 .7Hz, 1H), 7 .51-7 .49(m, 1H), 7 .34-7 .30(m, 2H), 7 .02(d, J = 8 .2Hz, 2H), 6 .68(d, J = 8 .3Hz, 2H), 4 .61(d, J = 6 .2Hz, 2H), 2 .24(s, 3H).

^{13}C NMR (151MHz, CDCl3) δ164 .26, 150 .83, 145 .82, 145 .80, 144 .53, 140 .87, 129 .83, 127 .91, 125 .00, 124 .39, 119 .93, 113 .37, 110 .62, 42 .25, 20 .39.

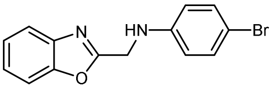

5i

N-(benzo[d]oxazol-2-ylmethyl)-4-bromoaniline (5i)

A mixture of 4-bromoaniline (18.0 mmol) and 2-(chloromethyl)benzo[d]oxazole (18.0 mmol) was

dissolved in DMF (10 mL). Potassium carbonate (0.90 g, 6.48 mmol) and KI was added to the mixture. The solution at room temperature was stirred for 12 hours. And then the solution was poured into water and extracted with ethyl acetate. The combined organic layers were washed with water and brine, dried over Na2SO4 and concentrated in vacuo. The residue was purified by column chromatography on silica gel (petroleum ether : ethyl acetate = 4:1) to give compound **5i**.

^{1}H NMR(600MHz, CDCl3) δ7 .72(dd, J = 6 .3, 2 .8Hz, 1H), 7 .53(dd, J = 6 .3, 2 .9Hz, 1H), 7 .36-7 .34(m, 2H), 7 .30(d, J = 8 .8Hz, 2H), 6 .65(d, J = 8 .8Hz, 2H), 4 .61(d, J = 6 .0Hz, 2H).

^{13}C NMR (151MHz, CDCl3) δ163 .57, 150 .83, 145 .82, 140 .75, 132 .38, 132 .07, 125 .18, 124 .52, 119 .98, 116 .66, 114 .78, 110 .64, 110 .39, 77 .20, 76 .99, 76 .78, 41 .83, 0 .03.

Supplementary Table 2. In vitro P2Y14R affinity of compounds 5a-5i (IC50, nM)

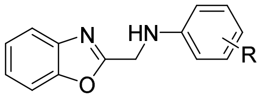

compds	R	IC50
5a	H	5.36
5b	3-Br	0.33
5c	3-Cl	12.39
5d	3-OCH_3	2.46
5e	2-Br	5.12
5f	4-Cl	1.26
5g	4-Br	7.78
5h	4-CH_3	2.74
5i	4-Br	13.32

Spectral data

^{13}C NMR spectra of HDL16

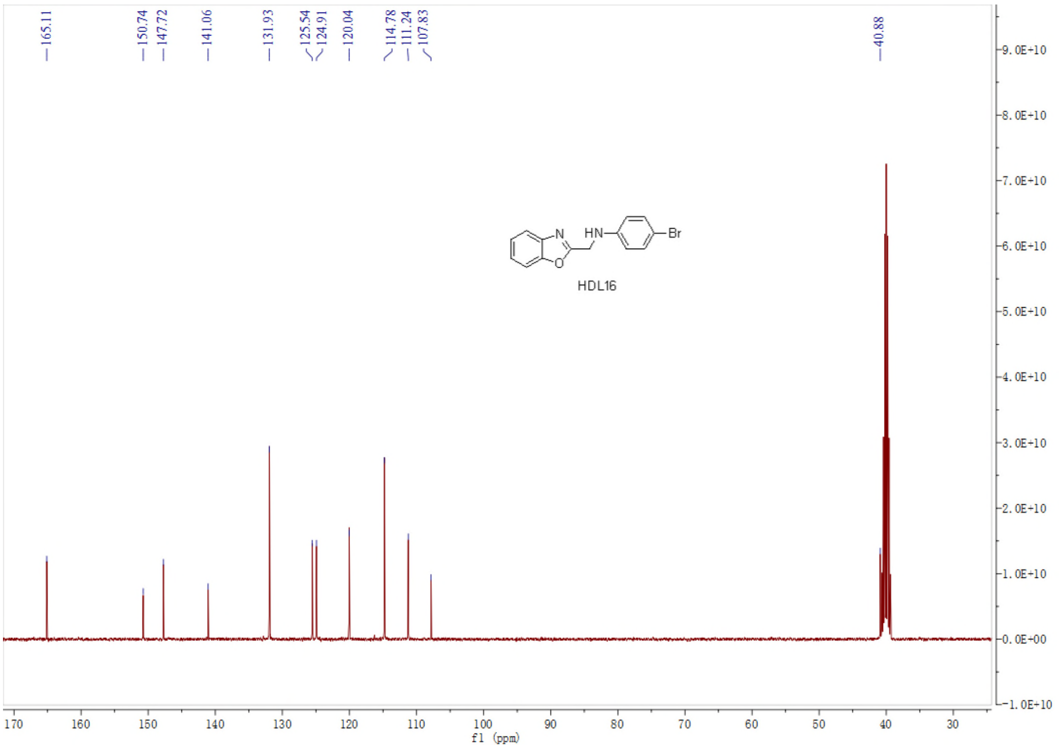

^{1}H NMR spectra of HDL16

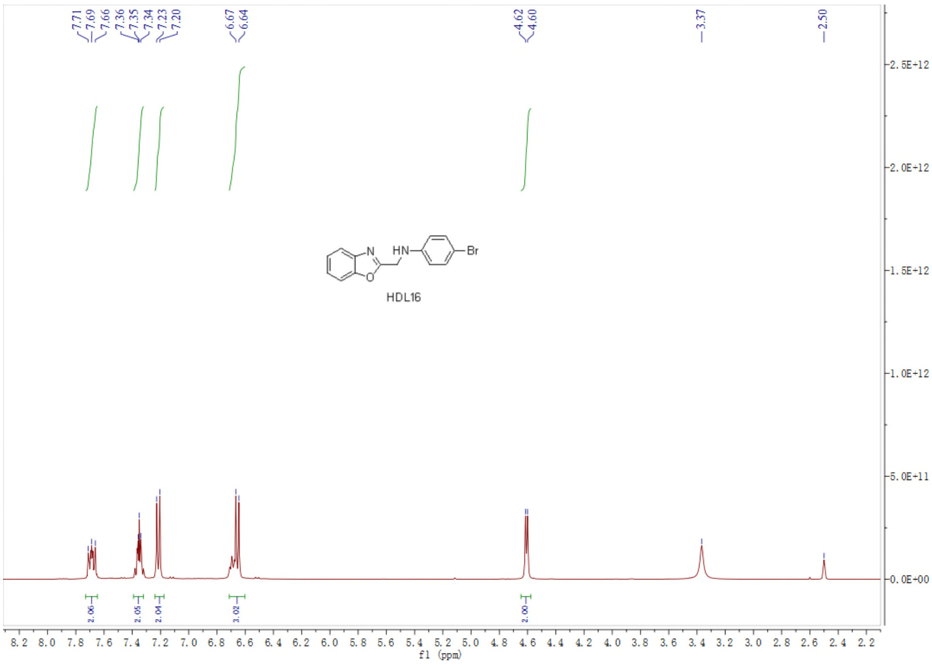

HRMS spectra of HDL16

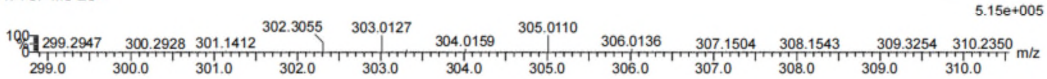

Elemental Composition Report Page 1

Single Mass Analysis
Tolerance = 5.0 PPM / DBE: min = -1.5, max = 50.0
Element prediction: Off
Number of isotope peaks used for i-FIT = 3

Monoisotopic Mass, Even Electron Ions
84 formula(e) evaluated with 1 results within limits (up to 50 best isotopic matches for each mass)
Elements Used:
C: 0-14 H: 0-12 B: 0-1 N: 0-2 O: 0-1 Na: 0-1 K: 0-1 Br: 0-1

HDL-16 2 (0.062)
1: TOF MS ES+

Minimum:				-1.5				
Maximum:		5.0	5.0	50.0				
Mass	Calc. Mass	mDa	PPM	DBE	i-FIT	Norm	Conf(%)	Formula
303.0127	303.0133	-0.6	-2.0	9.5	1020.1	n/a	n/a	C14 H12 N2 O Br

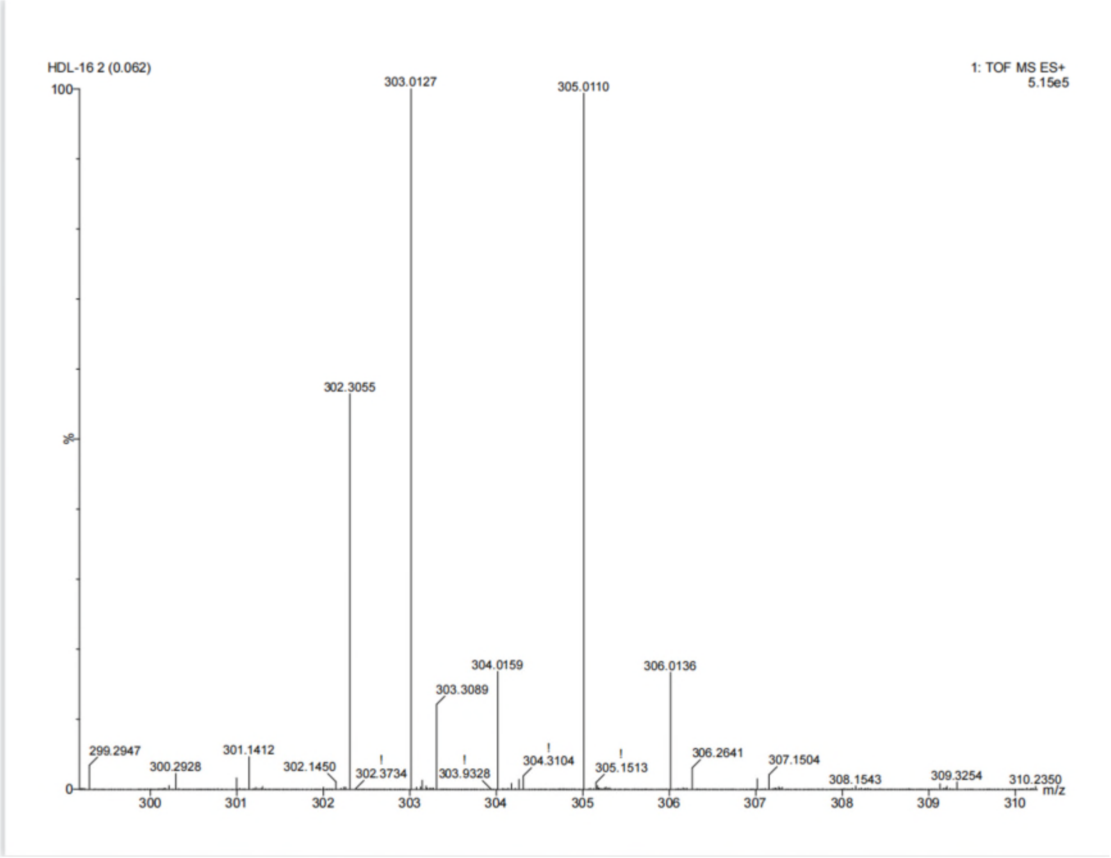
HDL-16 2 (0.062)
1: TOF MS ES+
5.15e5
303.0127
305.0110
302.3055
304.0159
306.0136
303.3089
299.2947
300.2928
301.1412
302.1450
302.3734
303.9328
304.3104
305.1513
306.2641
307.1504
308.1543
309.3254
310.2350
%
m/z
