# Supplementary material for: Targeting P2Y14R protects against necroptosis of intestinal epithelial cells through PKA/CREB/RIPK1 axis in ulcerative colitis
Source: Nat Commun. 2024 Mar 7;15:2083. doi: 10.1038/s41467-024-46365-x (PMC10920779; doi:10.1038/s41467-024-46365-x)
Supplement: Supplementary file 3 — Reporting Summary [file 41467_2024_46365_MOESM3_ESM.pdf]

nature portfolio

Corresponding author(s): Professor Qinghua Hu

Last updated by author(s): Jan 22, 2024

Reporting Summary

Nature Portfolio wishes to improve the reproducibility of the work that we publish. This form provides structure for consistency and transparency in reporting. For further information on Nature Portfolio policies, see our Editorial Policies and the Editorial Policy Checklist.

Statistics

For all statistical analyses, confirm that the following items are present in the figure legend, table legend, main text, or Methods section.

n/a	Confirmed	
☐	☒	The exact sample size (*n*) for each experimental group/condition, given as a discrete number and unit of measurement
☐	☒	A statement on whether measurements were taken from distinct samples or whether the same sample was measured repeatedly
☐	☒	The statistical test(s) used AND whether they are one- or two-sided<br>*Only common tests should be described solely by name; describe more complex techniques in the Methods section.*
☒	☐	A description of all covariates tested
☐	☒	A description of any assumptions or corrections, such as tests of normality and adjustment for multiple comparisons
☐	☒	A full description of the statistical parameters including central tendency (e.g. means) or other basic estimates (e.g. regression coefficient) AND variation (e.g. standard deviation) or associated estimates of uncertainty (e.g. confidence intervals)
☐	☒	For null hypothesis testing, the test statistic (e.g. *F*, *t*, *r*) with confidence intervals, effect sizes, degrees of freedom and *P* value noted<br>*Give P values as exact values whenever suitable.*
☒	☐	For Bayesian analysis, information on the choice of priors and Markov chain Monte Carlo settings
☒	☐	For hierarchical and complex designs, identification of the appropriate level for tests and full reporting of outcomes
☒	☐	Estimates of effect sizes (e.g. Cohen's *d*, Pearson's *r*), indicating how they were calculated

Our web collection on statistics for biologists contains articles on many of the points above.

Software and code

Policy information about availability of computer code

Data collection	Images were collected using cellSens (version 3.2) to operatec, Images were collected using Image-Pro Plus software (version 6.0) to operate BX53 microscope (Olympus), ZEN imaging software version 2012 to operate confocal scanning microscope LSM 800 (Zeiss). Immunoblot Images were taken using ChemiScope 6000 software to operate ChemiSignal ECL Plus (CLINX). qPCR were performed QuantStudio "Design & Analysis" Software to Biosystems QuantStudio 1. UV absorption, fluorescence, and chemiluminescence were measured by multi-mode microplate reader Cytation5 (BioTek). NDP scan 3.2.6 to operate NanoZoomer S60 C13210-01 (Hamamatsu).
Data analysis	All statistical analysis were performed on Graphpad Prism (version 9.4.0). Image j was used to analyze the fluorescent images and Immunoblot images.

For manuscripts utilizing custom algorithms or software that are central to the research but not yet described in published literature, software must be made available to editors and reviewers. We strongly encourage code deposition in a community repository (e.g. GitHub). See the Nature Portfolio guidelines for submitting code & software for further information.

Data

Policy information about availability of data

All manuscripts must include a data availability statement. This statement should provide the following information, where applicable:

- Accession codes, unique identifiers, or web links for publicly available datasets
- A description of any restrictions on data availability
- For clinical datasets or third party data, please ensure that the statement adheres to our policy

All data generated or analyzed during this study are included in this published article (and its supplementary datasets files). Source data are provided with this paper. All the datasets in this study are existing published and are available via the NCBI website, including Gene Expression Omnibus (GSE) accession number: GSE38713 (https://www.ncbi.nlm.nih.gov/geo/query/acc.cgi?acc=GSE38713), GSE75214 (https://www.ncbi.nlm.nih.gov/geo/query/acc.cgi?acc=GSE75214), GSE6879 (https://www.ncbi.nlm.nih.gov/geo/query/acc.cgi?acc=GSE6879), GSE117993 (https://www.ncbi.nlm.nih.gov/geo/query/acc.cgi?acc=GSE117993), GSE126124 (https://www.ncbi.nlm.nih.gov/geo/query/acc.cgi?acc=GSE126124).

Research involving human participants, their data, or biological material

Policy information about studies with human participants or human data. See also policy information about sex, gender (identity/presentation), and sexual orientation and race, ethnicity and racism.

Reporting on sex and gender	This study was approved by the Institutional Review Board of Nanjing First Hospital and informed consent was obtained from the study participants. The age and gender of the patients have been documented.
Reporting on race, ethnicity, or other socially relevant groupings	All the patients are of Han ethnicity, which is the largest ethnic group in China.
Population characteristics	For immunofluorescence in Figure 1b, Healthy control obtained from paracancerous tissues of descending colon cancer patient (female, age: 35), Ulcerative colitis tissues obtained from Ulcerative colitis patient (male, age: 44), Crohn's Disease patient tissues obtained from Crohn's Disease patient (male, age 15).
Recruitment	Patients were enrolled in by gastroenterologist of Nanjing First Hospital with informed consent from patients.
Ethics oversight	This study was approved by the Institutional Review Board of Nanjing First Hospital (KY20180604-05-KS-01).

Note that full information on the approval of the study protocol must also be provided in the manuscript.

Field-specific reporting

Please select the one below that is the best fit for your research. If you are not sure, read the appropriate sections before making your selection.

[x] Life sciences   [ ] Behavioural & social sciences   [ ] Ecological, evolutionary & environmental sciences

For a reference copy of the document with all sections, see nature.com/documents/nr-reporting-summary-flat.pdf

Life sciences study design

All studies must disclose on these points even when the disclosure is negative.

Sample size	Sample size in this study were based on previous studies with similar experiments, e.g. PMID: 37669965 (Li X, et al. 2020 Nat Commun.), e.g. PMID: 35974017 (Dong L, et al. 2022 Nat Commun.), PMID: 35974017 (Patankar JV, et al. 2021 Nat Cell Biol.). Sample size for each experiments have indicated in figure legend, all data are representative of at least three independent experiments. For animal experiments Sample size was chosen to ensure reproducibility of the experiments in accordance with the replacement, reduction and refinement principles of animal ethics regulation.
Data exclusions	No samples were excluded from the analyses.
Replication	All experimental findings were reliably reproduced. All in vitro and in vivo experiments were carried out with at least 3 biological replicates for each experimental group. Biological replicates are indicated within figure legends.
Randomization	All samples were randomly allocated into experimental groups.
Blinding	No blinding was used throughout experiments. The Investigators were not blinded to allocation during experiments and outcome assessment, and the data analyses were based on objectively measurable data.

Reporting for specific materials, systems and methods

We require information from authors about some types of materials, experimental systems and methods used in many studies. Here, indicate whether each material, system or method listed is relevant to your study. If you are not sure if a list item applies to your research, read the appropriate section before selecting a response.

Materials & experimental systems

n/a	Involved in the study
☐	☒ Antibodies
☐	☒ Eukaryotic cell lines
☒	☐ Palaeontology and archaeology
☐	☒ Animals and other organisms
☒	☐ Clinical data
☒	☐ Dual use research of concern
☒	☐ Plants

Methods

n/a	Involved in the study
☒	☐ ChIP-seq
☒	☐ Flow cytometry
☒	☐ MRI-based neuroimaging

Antibodies

Antibodies used

RIPK1 Rabbit Monoclonal Antibody (17519-1-AP, Proteintech, 1:1000 dilution)
MLKL Rabbit Monoclonal Antibody(GTX107538-100, GeneTex, 1:1000)
RIPK3 Rabbit Monoclonal Antibody(17563-1-AP, Proteintech, 1:1000)
p-MLKL Rabbit Monoclonal Antibody (EPR9514, Abcam, 1:1000)
PKA Rabbit Monoclonal Antibody (bs-0520R, Bioss,1:1000)
p-PKA Rabbit Monoclonal Antibody (#9621, Cell signaling, 1:1000)
CREB Rabbit Monoclonal Antibody (AF6188, Affinity, 1:1000)
p-CREB Rabbit Monoclonal Antibody (AF3189, Affinity, 1:1000)
GPR105 Rabbit Monoclonal Antibody (bs-12028R, Bioss, 1:1000)
GAPDH Rabbit Monoclonal Antibody (BS-2188R, Bioss, 1:1000)
Claudin-1 Rabbit Monoclonal Antibody (proteintech, 28674-1-AP, 1:300)
Occludin Rabbit Monoclonal Antibody (bioss, bs-10011R, 1:300)
ZO-1 Rabbit Monoclonal Antibody(Affinity, AF5145, 1:300)
EpCAM Mouse Monoclonal Antibody (AiFang, AF04654, 1:300)
P2Y14R Rabbit Monoclonal Antibody (Invitrogen, PAS-103202, 1:300)
Cleaved Caspase-3 (Asp175) (5A1E) RabbitMonoclonal Antibody (Cell signaling, #9664, 1:1000)
Goat Anti-Rabbit IgG H&L Antibody (bioss, Bs-0295G, 1:8000)
Goat Anti-Mouse IgG H&L Antibody (bioss, Bs-0296G, 1:8000)
Goat Anti-Mouse IgG H&L (Alexa Fluor® 555) (ab150114, Abcam, 1:500)
Goat Anti-Rabbit IgG H&L (Alexa Fluor® 488) (ab150077, Abcam, 1:300)

Validation

RIPK1 Rabbit Monoclonal Antibody (17519-1-AP, Proteintech, 1:1000 dilution)
https://www.ptgcn.com/products/RIPK1-Specific-Antibody-17519-1-AP.html
MLKL Rabbit Monoclonal Antibody(GTX107538-100, GeneTex, 1:1000)
http://www.neobioscience.com/search.aspx?TypeId=198&FId=t3:198:3&keyword=GTX107538
RIPK3 Rabbit Monoclonal Antibody(17563-1-AP, Proteintech, 1:1000)
https://www.ptgcn.com/products/RIPK3-Antibody-17563-1-AP.html
p-MLKL Rabbit Monoclonal Antibody (EPR9514, Abcam, 1:1000)
https://www.abcam.cn/products/primary-antibodies/mlkl-phospho-s358-antibody-epr9514-ab187091.html
PKA Rabbit Monoclonal Antibody (bs-0520R, Bioss,1:1000)
http://www.bioss.com.cn/prolook_03.asp?id=AF08169606000709&pro37=1
p-PKA Rabbit Monoclonal Antibody (#9621, Cell signaling, 1:1000)
https://www.cellsignal.cn/products/primary-antibodies/phospho-ser-thr-pka-substrate-antibody/9621?site-search-type=Products&N=4294956287&Ntt=%239621&fromPage=plp&_requestid=115262
CREB Rabbit Monoclonal Antibody (AF6188, Affinity, 1:1000)
https://www.affbiotech.cn/goods-1819-AF6188-CREB_Antibody.html
p-CREB Rabbit Monoclonal Antibody (AF3189, Affinity, 1:1000)
https://www.affbiotech.cn/goods-1367-AF3189-Phospho_CREB_Ser133_Antibody.html
GPR105 Rabbit Monoclonal Antibody (bs-12028R, Bioss, 1:1000)
http://www.bioss.com.cn/prolook_03.asp?id=AF08169606015500&pro37=1
GAPDH Rabbit Monoclonal Antibody (BS-2188R, Bioss, 1:1000)
http://www.bioss.com.cn/prolook_03.asp?id=AF08169606002341&pro37=1
Claudin-1 Rabbit Monoclonal Antibody (bioss, bs-10011R, 1:1000)
https://www.ptgcn.com/products/Claudin-1-Antibody-28674-1-AP.htm
Occludin Rabbit Monoclonal Antibody (bioss, DF6919, 1:1000)
http://www.bioss.com.cn/prolook_03.asp?id=AF08169606019641&pro37=1
ZO-1 Rabbit Monoclonal Antibody(Affinity, AF5145, 1:1000)
https://www.affbiotech.cn/goods-4452-AF5145-ZO_1_Antibody.html
EpCAM Mouse Monoclonal Antibody (AiFang, AF04654, 1:1000)

http://www.afantibody.cn/search.aspx?Key=AF04654
P2Y14R Rabbit Monoclonal Antibody (Invitrogen, PAS-103202, 1:1000)
https://www.thermofisher.cn/cn/zh/antibody/product/P2Y14-Antibody-Polyclonal/PA5-103202
Cleaved Caspase-3 (Asp175) (5A1E) RabbitMonoclonal Antibody (Cell signaling, #9664, 1:1000)
https://www.cellsignal.cn/products/primary-antibodies/cleaved-caspase-3-asp175-5a1e-rabbit-mab/9664
Goat Anti-Rabbit IgG H&L Antibody (bioss, Bs-0295G, 1:8000)
http://www.bioss.com.cn/prolook_03.asp?id=AF08169606003735&pro37=3
Goat Anti-Mouse IgG H&L Antibody (bioss, Bs-0296G, 1:8000)
http://www.bioss.com.cn/prolook_03.asp?id=AF08169606003738&pro37=3
Goat Anti-Mouse IgG H&L (Alexa Fluor® 555) (ab150114, Abcam, 1:500)
https://www.abcam.cn/products/secondary-antibodies/goat-mouse-igg-hl-alexa-fluor-555-ab150114.html
Goat Anti-Rabbit IgG H&L (Alexa Fluor® 488) (ab150077, Abcam, 1:300)
https://www.abcam.cn/products/secondary-antibodies/goat-rabbit-igg-hl-alexa-fluor-488-ab150077.html

Eukaryotic cell lines

Policy information about cell lines and Sex and Gender in Research

Cell line source(s)	HT-29 cells and HCT-116 cells were purchased from the BeNa Culture Collection Co, ltd. HEK293 cells stably expressing the hP2Y14R (P2Y14-HEK293 cells) were purchased from Keygen Biotech Co, ltd.<br>HT-29 cell line was isolated from the primary tumor derived from a 44-year-old white female patient with colorectal adenocarcinoma; HCT-116 cell line was derived from a 48-year-old male patient with colon cancer; HEK293 cell line was derived from human embryonic kidney cell.<br>Colonic organoids were collected and cultured from P2Y14R fl/fl and P2Y14R ΔIEC mice and the protocols were according to the previous publication [PMID: 35974017]. The sex of mice did not affect the phenotype of colonic organoids.
Authentication	These cell lines were authenticated by the supplier using STR analysis
Mycoplasma contamination	No mycoplasma contamination was found
Commonly misidentified lines (See ICLAC register)	These all cell lines that we used were not listed in commonly misidentified lines in ICLAC register

Animals and other research organisms

Policy information about studies involving animals; ARRIVE guidelines recommended for reporting animal research, and Sex and Gender in Research

Laboratory animals	The P2Y14Rfl/fl, Villin-cre, Lyz2-cre and wild-type (WT) mice with C57BL/6J background were purchased from Gempharmatech (GemPharmatech Co., Ltd) and bred onsite to generate animals for experimentation. All mice were housed in a controlled environment (20 ± 2°C, 40–60% humidity, 12-hour/12-hour light/dark cycle), where they were maintained on a standard chow diet (1010088, 1010083, Jiangsu Xietong Pharmaceutical Bio-engineering Co., Ltd.) with free access to water. Male, 7-8-week-old, P2Y14Rfl/fl Vil-cre (P2Y14RΔIEC), P2Y14Rfl/fl Lyz2-cre and WT mice with C57BL/6J background were used to establish experimental (acute/chronic) colitis model.
Wild animals	The study did not involve wild animals.
Reporting on sex	Only male mice were used for all the animal assay as the sex of mice did not affect the phenotype of DSS-induced IBD model, and single sex animals were used to reduced experiments variables.
Field-collected samples	The study did not involve samples collected from the field.
Ethics oversight	All animal experiments were performed in conformity with the Guide for the Care and Use of Laboratory Animals (NIH publication No. 85-23, 1996 revision) and approved by the China Pharmaceutical University Committee for Laboratory Animal Use.

Note that full information on the approval of the study protocol must also be provided in the manuscript.
